# *KRAS* and *BRAF* somatic mutations in colonic polyps and the risk of metachronous neoplasia

**DOI:** 10.1371/journal.pone.0184937

**Published:** 2017-09-27

**Authors:** Miriam Juárez, Cecilia Egoavil, María Rodríguez-Soler, Eva Hernández-Illán, Carla Guarinos, Araceli García-Martínez, Cristina Alenda, Mar Giner-Calabuig, Oscar Murcia, Carolina Mangas, Artemio Payá, José R. Aparicio, Francisco A. Ruiz, Juan Martínez, Juan A. Casellas, José L. Soto, Pedro Zapater, Rodrigo Jover

**Affiliations:** 1 Research Laboratory, Alicante University General Hospital, Alicante Institute for Health and Biomedical Research (ISABIAL-FISABIO Foundation), Alicante, Spain; 2 Department of Pathology, Alicante University General Hospital, Alicante Institute for Health and Biomedical Research (ISABIAL-FISABIO Foundation), Alicante, Spain; 3 Service of Digestive Medicine, Alicante University General Hospital, Alicante Institute for Health and Biomedical Research (ISABIAL-FISABIO Foundation), Alicante, Spain; 4 Molecular Genetics Laboratory, Elche University General Hospital, Alicante Institute for Health and Biomedical Research (ISABIAL-FISABIO Foundation), Elche, Spain; 5 Clinical Pharmacology Department, Alicante University General Hospital, Alicante Institute for Health and Biomedical Research (ISABIAL-FISABIO Foundation), Alicante, Spain; University of Munich, GERMANY

## Abstract

**Background & aims:**

High-risk features of colonic polyps are based on size, number, and pathologic characteristics. Surveillance colonoscopy is often recommended according to these findings. This study aimed to determine whether the molecular characteristics of polyps might provide information about the risk of metachronous advanced neoplasia.

**Methodology:**

We retrospectively included 308 patients with colonic polyps. A total of 995 polyps were collected and tested for somatic *BRAF* and *KRAS* mutations. Patients were classified into 3 subgroups, based on the polyp mutational profile at baseline, as follows: non-mutated polyps (Wild-type), at least one *BRAF*-mutated polyp, or at least one *KRAS*-mutated polyp. At surveillance, advanced adenomas were defined as adenomas ≥ 10 mm and/or with high grade dysplasia or a villous component. In contrast, advanced serrated polyps were defined as serrated polyps ≥ 10 mm in any location, located proximal to the splenic flexure with any size or with dysplasia.

**Results:**

At baseline, 289 patients could be classified as wild-type (62.3%), *BRAF* mutated (14.9%), or *KRAS* mutated (22.8%). In the univariate analysis, KRAS mutations were associated with the development of metachronous advanced polyps (OR: 2.36, 95% CI: 1.22–4.58; *P* = 0.011), and specifically, advanced adenomas (OR: 2.42, 95% CI: 1.13–5.21; *P* = 0.023). The multivariate analysis, adjusted for age and sex, also showed associations with the development of metachronous advanced polyps (OR: 2.27, 95% CI: 1.15–4.46) and advanced adenomas (OR: 2.23, 95% CI: 1.02–4.85).

**Conclusions:**

Our results suggested that somatic *KRAS* mutations in polyps represent a potential molecular marker for the risk of developing advanced neoplasia.

## Introduction

Colorectal cancer (CRC) is a heterogeneous group of diseases that can develop through distinct pathways involving different genetic and epigenetic changes [1]. Conventional adenoma is the principal precursor of CRC [2] through the classical adenoma-carcinoma pathway, which represents around 75% of these tumours [3]. On the other hand, the serrated pathway has emerged as the second most significant pathway; it represents the progression of serrated lesions to CRC [4], and it is responsible for up to 20–30% of all CRCs [1, 5]. Colonoscopy is considered the main method for detecting and removing precursor lesions, through screening and surveillance for CRC [6]. Surveillance colonoscopy is often recommended according to the characteristics of polyps, mainly the size and number, determined at a baseline colonoscopy [7]. Although the number of strategies for screening could increase, due to emerging technologies in molecular marker applications [8–9], to date, no molecular information has been useful in predicting whether new lesions will be detected at follow-up.

*KRAS* and *BRAF* belong to the intracellular RAS/RAF/MEK/mitogen-activated protein kinase (MAPK) cascade, which mediates cellular responses to growth signals. Activating *KRAS* mutations occur in 30–50% of CRCs [10]. These mutations occur during the early to advanced stages of the polyp-to-carcinoma sequence. On the other hand, *BRAF* is mutated very early in the serrated pathway, and approximately 10% of all CRCs carry an activating mutation in this oncogene [11].

The present study aimed to determine whether molecular characteristics of polyps, specifically somatic *BRAF* and *KRAS* mutations, might provide information about the risk of developing metachronous advanced neoplasia during follow-up for patients diagnosed with polyps.

## Materials and methods

### Patients and subgroup classification

We retrospectively recruited patients diagnosed with polyps in a colonoscopic examination between the years 2007 and 2009 at the Hospital General Universitario of Alicante. All the included patients had at least one surveillance colonoscopy performed more than 6 months after the baseline examination. Data on surveillance colonoscopies were collected until December 2014. Colonoscopy was performed either on the basis of symptoms or as a follow-up surveillance after a CRC or adenoma excision. In these patients the first surveillance colonoscopy performed during the period of the study has been considered as the baseline colonoscopy in terms of subsequent follow-up. Clinicopathological information and patient personal history were also collected. Patients were excluded when they were diagnosed of CRC at the inclusion in the study or they were previously diagnosed with polyposis syndrome, Lynch syndrome, or inflammatory bowel disease.

This study was approved by the Ethics Committee of the Hospital General Universitario of Alicante, and all clinical data of patients were anonymized.

### Samples

A total of 995 polyps from 308 patients were collected for histological and molecular analysis. These polyps were obtained from both, the baseline and subsequent surveillance colonoscopies during the period of the study. All polyps were removed endoscopically. Endoscopy and the corresponding histopathology reports were reviewed to collect information about the number, size, morphology, distribution, and pathology of polyps.

The polyps were categorised as conventional adenomas and serrated lesions. Conventional adenomas were differentiated as tubular, tubulovillous, or villous adenomas, according to standard criteria [12]. Serrated lesions were classified as hyperplastic polyps (HP), sessile serrated adenomas (SSA), traditional serrated adenomas (TSA) [13]. HPs were also classified as microvesicular type, globet type and mucine-poor hiperplastic polyps. A review of all polyps was performed by two experienced pathologists in our group (C.E. and C.A.), to avoid inter-observer errors.

Polyps were considered to be located in the right colon when they were in the ascending colon, transverse colon, or caecum. They were considered to be located in the left colon when they were in the descending colon (including the splenic flexure), sigmoid colon, or rectum.

Polyps were classified according to high risk features. Advanced adenomas were defined as adenomas ≥ 10 mm and/or with high grade dysplasia or a villous component. Advanced serrated lesions were defined as serrated lesions ≥ 10 mm in any location, located proximal to the splenic flexure with any size or with dysplasia [14–16].

For each polyp, samples from paraffin-embedded tissue were microdissected in ten, 5-μm-thick sections. Sample DNA was extracted with the QIAamp DNA Investigator kit (QIAGEN, Hilden Germany) and with the E.Z.N.A Forensic DNA kit (OMEGA Bio-tek, USA), according to manufacturer´s protocols.

### Somatic *BRAF* and *KRAS* analysis

#### All polyps were tested for somatic *BRAF* and *KRAS* mutations

*BRAF* mutations at codon 600 (V600E) were identified with real time PCR (ABI PRISM 7500, Applied Biosystems, Foster City, CA, USA), based on the allelic discrimination method (Applied Biosystems, Foster City, CA, USA). We used specific TaqMan probes, as previously described by Benlloch et al. [17].

*KRAS* mutations at exon 2, which included codons 12 and 13, were identified with DNA Sanger sequencing (ABI3500 Genetic Analyzer, Applied Biosystems), as previously described [18].

Patients were classified into 3 subgroups, based on the mutational profile of their polyps at a baseline colonoscopy, as follows: 1) wild-type group (WT); patients with polyps at a baseline colonoscopy with no mutation in either the *BRAF* or *KRAS* gene; 2) *BRAF* group: patients with at least one *BRAF*-mutated polyp; and 3) *KRAS* group: patients with at least one *KRAS*-mutated polyp at a baseline colonoscopy. Patients with both *BRAF* and *KRAS* somatic mutation found at their polyps were excluded.

### Statistical analysis

Data analyses were carried out to determine statistical significance with SPSS software (SPSS 19.0, Chicago, IL, USA). Parametric continuous variables are reported as the mean ± standard deviation (SD); nonparametric continuous variables are reported as the median and interquartile range (IQR). On the other hand, categorical variables are reported as frequencies or percentages. Differences between samples were determined with the Student *t* test or ANOVA analyses for parametric quantitative data. Statistical differences between the groups were analyzed using a chi-squared method for categorical data followed by Yates correction or Fisher’s exact test, where appropriate.

We included univariate and multivariate logistic regression models to determine the association between the detection of advanced lesions at surveillance and the clinical and the molecular characteristics of lesions at baseline. The multivariate analysis was performed after adjusting for the sex and age of patients. Also variables found to be significant in the univariate analyses were included in the multivariate analysis. Results are expressed as the odds ratios (OR) with 95% confidence intervals (95% CI).

Kaplan-Meier survival curves were compared with the log-rank test. *P*-values less than 0.05 were considered significant.

## Results

### Pathological and molecular characteristics of polyps

Nine hundred ninety-five polyps from 308 patients were reviewed for pathology and evaluated with molecular analyses. A total of 661 polyps (66.4%) were categorised as conventional adenomas (tubular adenoma, n = 593; tubulovillous, n = 63; villous adenoma, n = 5); and 334 polyps (33.6%) were categorised as serrated lesions (HP, n = 281; SSA, n = 45; TSA, n = 8). A total of 263 conventional adenomas (39.8%) were considered advanced, and a total of 87 serrated lesions were classified as advanced serrated lesions (26.0%).

The characteristics of polyps, based on their mutational profiles, are shown in Table 1. A total of 665 polyps were WT (72.0%), 124 had *BRAF* mutations (13.4%) and 135 had *KRAS* mutations (14.6%). As expected, *BRAF* mutations were extremely rare in adenomas; they were found in only 0.8% of all adenomas, and in 39.4% of serrated lesions. On the other hand, *KRAS* mutations were found in 11.6% of adenomas and 20.9% of serrated lesions. *BRAF* and *KRAS* mutated polyps were more frequently found in the left colon than in the right colon (*P*<0.0001; left colon 16.1% *BRAF* mutations and 17.2% *KRAS* mutations; right colon: 8.1% *BRAF* mutations and 9.4% *KRAS* mutations). Among advanced lesions, we observed that no advanced adenoma carried a *BRAF* mutation, but 45% of advanced serrated lesions harboured this mutation. Moreover, *KRAS* mutations were observed more frequently in advanced adenomas (22.0%) than in non-advanced adenomas (4.8%; *P*<0.0001). Polyps were more frequently larger than 10 mm in lesions with *KRAS* mutations (21.8% ≥10 mm and 11.3% <10 mm; *P*<0.0001). In addition, *KRAS* mutated polyps more frequently exhibited high grade dysplasia (low grade dysplasia: 11.1%; high grade dysplasia: 45.7%; *P*<0.0001).

**Table 1:** Molecular characteristics of polyps.

	WILD-TYPE 665 polyps	*BRAF* mutation 124 polyps	*KRAS* mutation 135 polyps	*P*-value
**HISTOLOGY**, n (%)				
**Adenoma**	**545 (87.6%)**	**5 (0.8%)**	**72 (11.6%)**	<0.001*
Tubular	509 (91.1%)	5 (0.9%)	45 (8.1%)	
Tubulovillous	32 (55.2%)	0	26 (44.8%)	
Villous	4 (80.0%)	0	1 (20.0%)	
**Serrated lesions**	**120 (39.7%)**	**119 (39.4%)**	**63 (20.9%)**	
Hyperplastic polyps	105 (41.8%)	99 (39.4%)	47 (18.7%)	**<0.001**^†^
-Microvesicular Type	101 (43.2%)	92 (39.3%)	17.5%)	
-Goblet Type	2 (25.0%)	1 (12.5%)	(62.5%)	
-Mucine-Poor Type	2 (22.2%)	6 (66.7%)	1 (11.1%)	
SSA	14 (32.6%)	19 (44.2%)	10 (23.3%)	
TSA	1 (12.5%)	1 (12.5%)	6 (75.0%)	
**LOCATION**, n (%)				
Right	245 (82.5%)	24 (8.1%)	28 (9.4%)	**<0.001**
Left	415 (66.7%)	100 (16.1%)	107 (17.2%)	
**SIZE**, n (%)				
<10 mm	444 (72.8%)	97 (15.9%)	69 (11.3%)	**<0.001**
≥10 mm	198 (70.7%)	21 (7.5%)	61 (21.8%)	
**GRADE OF DYSPLASIA**, n (%)				
High	19 (54.3%)	0	16 (45.7%)	**<0.001**
Low	540 (87.2%)	10 (1.6%)	69 (11.1%)	
**MORPHOLOGY**, n (%)				
Pedunculated	125 (75.3%)	8 (4.8%)	33 (19.9%)	**0.001**
Non-Pedunculated	327 (72.2%)	68 (15.0%)	58 (12.8%)	
**ADVANCED ADENOMAS**, n (%)				
Yes	191 (78.0%)	0	54 (22.0%)	**<0.001**
No	354 (93.9%)	5 (1.3%)	18 (4.8%)	
**ADVANCED SERRATED LESIONS**, n (%)				
Yes	31(38.8%)	36 (45.0%)	13 (16.3%)	0.4
No	89 (40.1%)	83 (37.4%)	50 (22.5%)	

Abbreviations: SSA, sessile serrated adenoma; TSA, traditional serrated adenoma

Statistically significant results are represented in bold.

* *P*-value is referred to comparison between adenomas and serrated lesions.

^**†**^ *P*-value is referred to comparison between the different types of serrated lesions.

### Clinical and molecular characteristics of patients and the risk of developing metachronous advanced lesions

We retrospectively analysed the relationship between the clinical and molecular characteristics of polyps and the risk of developing metachronous neoplasia in a cohort of 308 patients. The mean age at diagnosis was 61 years (SD, 11.95; range 26–86) and the proportion of men was 62.3%.

A total of 289 cases were classified according to the mutational profiles of their polyps at a baseline colonoscopy. Nineteen cases were excluded, because they had equal proportions of *KRAS* and *BRAF* mutations (10 cases) or their polyps were not evaluable for *BRAF* and *KRAS* mutations (9 cases). The mean follow-up time was 36.6 months (SD: 15.6), with a median of 36 months (IQR: 25–49). The mean number of polyps diagnosed at baseline was 2.73 (SD: 2.43), with a median of 2 (IQR: 1–3). A subset of the patients included in this study has been previously diagnosed of CRC (n = 44, 15.2%) and/or previous polyps (n = 75, 26%). For these patients the first surveillance colonoscopy performed during the period of the study has been considered as the baseline colonoscopy.

According to their mutational profiles at baseline, 180 (62.3%) patients did not have any mutation in these two markers (WT group), 43 patients (14.9%) displayed *BRAF* mutations (*BRAF* group), and 66 patients (22.8%) displayed *KRAS* mutations (*KRAS* group). Table 2 shows the baseline characteristics of patients, according to the mutational status of their polyps. We observed that patients with *KRAS* mutations were older, frequently had more than 3 adenomas, and their polyps were ≥10 mm.

**Table 2:** Clinical characteristics of patients, according the polyp mutational profile assessed at a baseline colonoscopy.

	WILD-TYPE GROUP	*BRAF* GROUP	*KRAS* GROUP	*P*-value
	n = 180	n = 43	n = 66	
**AGE**, mean(SD)	60.84 ±12.02	56.72±12.73	65.44±9.78	**0.001**
**SEX**, n(%)				
Male	110 (61.1%)	25 (13.9%)	45 (25.0%)	0.5
Female	70 (64.2%)	18 (16.5%)	21 (19.3%)	
**MONTHS OF FOLLOW-UP**, mean(SD)	35.77±15.17	37.19±16.93	38.36±16.06	0.5
**PERSONAL HISTORY OF CRC**, n(%)				
No (n = 245)	152 (62.0%)	35 (14.3%)	58 (23.7%)	0.6
Yes (n = 44)	28 (63.6%)	8 (18.2%)	8 (18.2%)	
**PREVIOUS POLYPS**, n(%)				
No (n = 214)	131 (61.2%)	31 (14.5%)	52 (24.3%)	0.6
Yes (n = 75)	49 (65.3%)	12 (16.0%)	14 (18.7%)	
**POLYP NUMBER**, n(%)				
<3	126 (70%)	26 (14.4%)	28 (15.6%)	**<0.001**
≥ 3	54 (49.5%)	17 (15.6%)	38 (34.9%)	
**ADENOMAS**, n(%)				
<3	162 (65.3%)	39 (15.7%)	47 (19.0%)	**0.001**
≥ 3	18 (43.9%)	4 (9.8%)	19 (46.3%)	
**SERRATED LESIONS**, n(%)				
<3	177 (63.2%)	40 (14.3%)	63 (22.5%)	0.1
≥ 3	3 (33.3%)	3 (33.3%)	3 (33.3%)	
**≥10 mm POLYPS**, n(%)				
No	95 (68.3%)	28 (20.1%)	16 (11.5%)	**<0.001**
Yes	85 (56.7%)	15 (10.0%)	50 (33.3%)	
**POLYPS IN THE RIGHT COLON**, n(%)				
No	107 (64.1%)	28 (16.8%)	32 (19.2%)	0.2
Yes	73 (59.8%)	15 (12.3%)	34 (27.9%)	
**POLYPS IN THE LEFT COLON**, n(%)				
No	35 (72.9%)	3 (6.3%)	10 (20.8%)	0.1
Yes	145 (60.2%)	40 (16.6%)	56 (23.2%)	

Abbreviations: CRC, colorectal cancer; SD, standard deviation.

Statistically significant results are represented in bold.

During surveillance, a total of 401 lesions and 1 CRC were found. Pathologically, 237 were conventional adenomas and 164 serrated lesions. We classified 53 as advanced adenomas and 43 as advanced serrated lesions. The mean number of polyps at follow-up colonoscopies was 2.20 (SD: 2.95) polyps, with a median of 1 (IQR: 0–3).

Among the 289 patients with lesions, we investigated the risk of developing metachronous lesions for the different molecular subtypes. A total of 179 patients (61.9%) developed polyps at surveillance, 36 patients developed advanced adenomas (12.5%) and 26 patients advanced serrated lesions (9.0%).

In the univariate analysis, only the presence of a *KRAS* mutation in the polyp at baseline was associated with developing metachronous advanced polyps of any type (OR: 2.36, 95% CI: 1.22–4.58; *P* = 0.011 vs. non-mutated), and more specifically, advanced adenomas (OR: 2.42, 95% CI: 1.13–5.21; *P* = 0.023 vs. non-mutated) (Table 3). None of the other baseline characteristics (age, sex, previous CRC, high grade dysplasia, or size larger than 10 mm) were related to the development of advanced lesions or advanced adenomas at follow-up (Table 3). The baseline characteristics related to the development of advanced serrated lesions were male sex, previous CRC, and large lesions (Table 3).

**Table 3:** Univariate logistic regression analysis of risk of developing advanced lesions at surveillance, according to molecular and clinical characteristics of patients at the baseline colonoscopy.

**BASELINE CHARACTERISTICS**	**Risk of developing the indicated lesion at follow-up surveillance**								
	**ADVANCED ADENOMAS**			**ADVANCED SERRATED LESIONS**			**ANY ADVANCED POLYP**		
	**n (%)**	**OR**	***P*-value**	**n (%)**	**OR**	***P*-value**	**n (%)**	**OR**	***P*-value**
		**(95% CI)**			**(95% CI)**			**(95% CI)**	
**CLASSIFICATION** (n)									
Wild Type (180)	18(10.0%)	1		15(8.3%)	1		28(15.6%)	1	
*BRAF* (43)	4(9.3%)	0.92	0.9	3(7.0%)	0.83	0.8	7(16.3%)	1.06	0.9
		(0.29–2.88)			(0.23–2.99)			(0.43–2.61)	
*KRAS* (66)	14(21.2%)	2.42	**0.023**	8(12.1%)	1.52	0.4	20(30.3%)	2.36	**0.011**
		(1.13–5.21)			(0.61–3.77)			(1.22–4.58)	
**AGE** (mean±SD)	63.81±10.34		0.2	59.31±10.76		0.4	62.25±10.55		0.5
**SEX** (n)									
Male (180)	25(13.9%)	1	0.3	21(11.7%)	1	**0.049**	40(22.2%)	1	0.1
Female (109)	11(10.1%)	0.69		5(4.6%)	0.36		15(13.8%)	0.56	
		(0.33–1.48)			(0.13–0.99)			(0.29–1.07)	
**PREVIOUS CRC**									
No	27(11.0%)	1	0.1	18(7.3%)	1	**0.025**	42(17.1%)	1	0.1
Yes	9(20.5%)	2.08		8(18.2%)	2.80		13(29.5%)	2.03	
		(0.90–4.78)			(1.14–6.92)			(0.98–4.19)	
**≥3 ADENOMAS**									
No	27(10.9%)	1	0.1	23(9.3%)	1	0.7	45(18.1%)	1	0.3
Yes	9(22.0%)	2.30		3(7.3%)	0.77		10(24.4%)	1.46	
		(0.99–5.34)			(0.22–2.70)			(0.66–3.18)	
**ADENOMAS HGD**									
No	32(12.1%)	1	0.5	24(9.1%)	1	0.9	50(18.9%)	1	0.8
Yes	4(16.7%)	1.46		2(8.3%)	0.91		5(20.8%)	1.13	
		(0.47–4.53)			(0.20–4.12)			(0.40–3.18)	
**BASELINE CHARACTERISTICS**	**Risk of developing the indicated lesion at follow-up surveillance**								
	**ADVANCED ADENOMAS**			**ADVANCED SERRATED LESIONS**			**ANY ADVANCED POLYP**		
	**n (%)**	**OR**	***P*-value**	**n (%)**	**OR**	***P*-value**	**n (%)**	**OR**	***P*-value**
		**(95% CI)**			**(95% CI)**			**(95% CI)**	
**VILLOUS COMPONENT**									
No	29(11.7%)	1	0.3	24(9.7%)	1	0.3	46(18.5%)	1	0.6
Yes	7(17.1%)	1.56		2(4.9%)	0.48		9(22.0%)	1.24	
		(0.63–3.83)			(0.11–2.11)			(0.55–2.77)	
**SIZE ≥10 mm ADENOMAS**									
No	17(10.4%)	1	0.2	20(12.3%)	1	**0.033**	33(20.2%)	1	0.6
Yes	19(15.1%)	1.53		6(4.8%)	0.36		22(17.5%)	0.83	
		(0.76–3.07)			(0.14–0.92)			(0.46–1.52)	
**ADVANCED SERRATED LESIONS AT BASELINE**									
No	30(11.8%)	1	0.4	23(9.1%)	1	0.9	47(18.5%)	1	0.5
Yes	6(17.1%)	1.55		3(8.6%)	0.94		8(22.9%)	1.31	
		(0.59–4.03)			(0.27–3.32)			(0.56–3.05)	
**LOCATION**									
**Right**									
No	17(10.2%)	1	0.2	14(8.4%)	1	0.7	29(17.4%)	1	0.4
Yes	19(15.6%)	1.63		12(9.8%)	1.19		26(21.3%)	1.29	
		(0.81–3.28)			(0.53–2.68)			(0.71–2.33)	
**Left**									
No	6(12.5%)	1	1.0	1(2.1%)	1	0.1	7(14.6%)	1	0.4
Yes	30(12.4%)	0.99		25(10.4%)	5.44		48(19.9%)	1.46	
		(0.39–2.54)			(0.72–41.15)			(0.62–3.45)	

Abbreviations: CI, confidence interval; CRC, Colorectal cancer; HGD, high grade dysplasia; OR, odds ratio; SD, standard deviation

Statistically significant results are represented in bold.

This association between advanced lesions of any type at follow-up and a KRAS mutation was also independently observed in the multivariate analysis, after adjusting for age and sex (OR: 2.27, 95% CI: 1.15–4.46). Moreover, KRAS mutations were specifically associated with the development of metachronous advanced adenomas (OR: 2.23, 95% CI: 1.02–4.85). None of the clinical characteristics that were significantly associated with the development of advanced serrated lesions in the univariate analysis were identified as independent predictors in the multivariate analysis (Table 4).

**Table 4:** Multivariate analysis of clinical and molecular characteristics of patients, adjusted for age and sex.

OUTCOME	OR	95% CI		*P*-value
Factors included in the analysis		Min.	Max.	
**ADVANCED ADENOMAS**				
Molecular Classification				
-Wild-type Group	1			
*-BRAF* Group	0.99	0.31	3.12	1.0
*-KRAS* Group	2.23	1.02	4.85	**0.044**
**ADVANCED SERRATED LESIONS**				
No Previous CRC	1			
Previous CRC	2.17	0.85	5.53	0.1
Adenomas Size <10 mm or no adenomas	1			
Adenomas Size ≥10 mm	0.40	0.15	1.05	0.1
**ANY ADVANCED POLYP**				
Molecular Classification				
-Wild-type Group	1			
*-BRAF* Group	1.08	0.43	2.71	0.9
*-KRAS* Group	2.27	1.15	4.46	**0.018**

Abbreviations: CI: confidence interval; CRC: Colorectal cancer; OR: odds ratio.

Statistically significant results are represented in bold.

**Fig 1:**
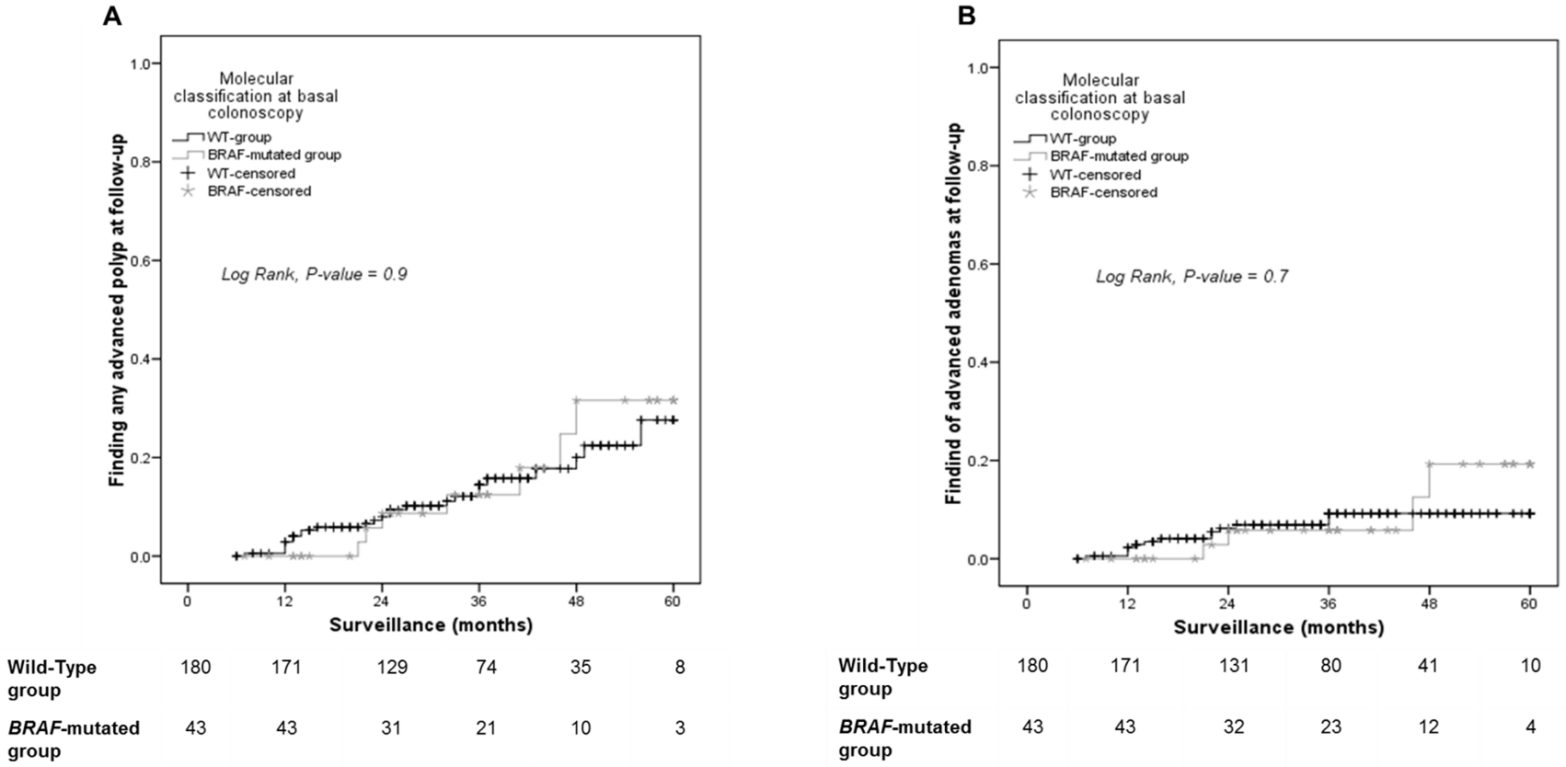
Risk of developing advanced polyps based on *BRAF* mutational status at baseline colonoscopy. Kaplan-Meier curves show the proportions of patients with WT or *BRAF*-mutated lesions that developed either (A) any advanced polyp or (B) advanced adenoma over time.

**Fig 2:**
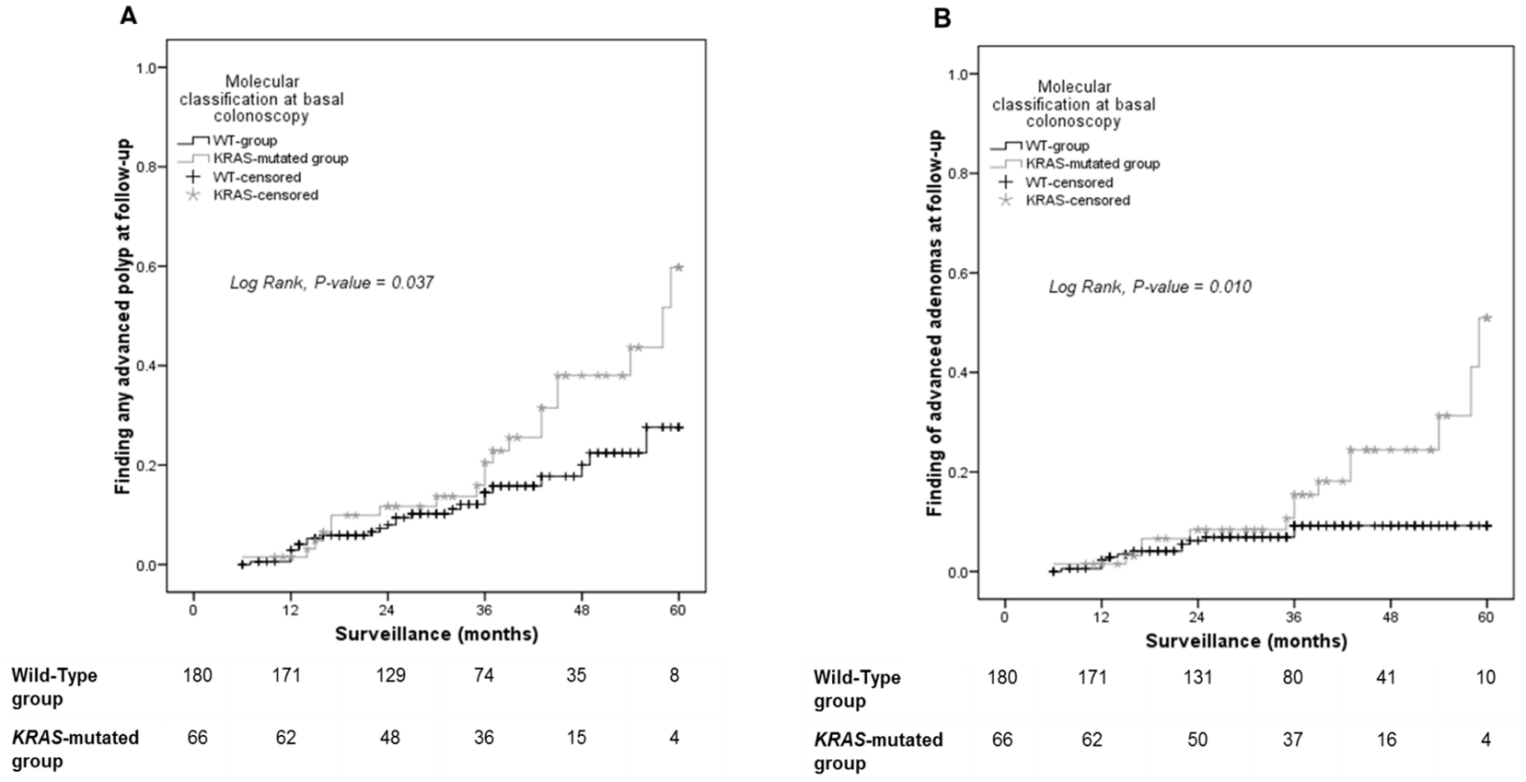
Risk of developing advanced polyps based on *KRAS* mutational status at baseline colonoscopy. Kaplan-Meier curves show the proportions of patients with WT or *KRAS*-mutated lesions that developed either (A) any advanced polyp or (B) advanced adenomas over time.

We performed Kaplan-Meier analyses to compare the risk of developing advanced polyps among patients with different baseline molecular characteristics. No differences were found between the *BRAF* and WT groups (log-rank for advanced polyps 0.9; log-rank for advanced adenomas 0.7) (Fig 1A and 1B). However, patients in the *KRAS* group developed advanced polyps (log-rank 0.037) and, more specifically, advanced adenomas (log-rank 0.010), at higher rates than patients in the WT group (Fig 2A and 2B).

## Discussion

The main finding of this study was that patients with at least one polyp that harboured a *KRAS* mutation were at higher risk of developing advanced polyps, specifically, advanced adenomas, compared to patients with polyps that harboured *BRAF* mutations or no mutation. Moreover, the *KRAS* mutation was an independent predictor of the development of advanced polyps and advanced adenomas, and it was a stronger predictor than other characteristics, like the size or number of lesions at baseline. These results established the potential utility of molecular markers for stratifying risk among patients with colonic polyps. Our findings suggested that the *KRAS* somatic mutation would be a useful marker for predicting the development of metachronous advanced neoplasia.

In the first part of our study, we classified a series of 995 polyps into 3 groups, according to their molecular characteristics: WT, *BRAF* mutated, and *KRAS* mutated. This classification was consistent with previously proposed CRC classifications [19–20] that emphasised the molecular background characteristics of colonic neoplasms. In our study, we linked precursor CRC lesions to a molecular pathway with the aim of determining whether this molecular signature could predict the development of advanced lesions at follow-up. As expected and according with previous studies, *BRAF* mutations were rarely found in conventional adenomas [21–24]. However, we found *BRAF* mutations in less than 40% of serrated lesions, which was clearly less frequent than previously reported for this type of polyps [25–26]. Our population was selected, given that we only included patients with a follow-up surveillance colonoscopy. Thus, our results could not be directly compared to results found in the general population. However, the potential bias of our patient selection would be towards selecting individuals with more advanced lesions.

Previous studies aimed to correlate advanced histological features or size with somatic *BRAF* or *KRAS* mutations to predict the risk of potential malignancy of polyps. Those studies observed a strong association between *KRAS* mutations, villous component, high-grade dysplasia and polyp size [23, 27–29], which suggested that *KRAS* mutations might increase the risk of progression in sporadic colorectal adenomas [27, 30]. Moreover, other studies have reported a significant association between *KRAS* mutations and advanced adenomas [31]. Our results were consistent with those previous studies; however, we also observed, that the presence of *KRAS* mutations in polyps at baseline could be an independent risk factor for the development of metachronous advanced lesions. In a similar previous study, Nusko G et al. did not find that *KRAS* mutations were a reliable prognostic factor of metachronous neoplasia [32]. However the number of patients included in this study was very small and only one index adenoma of each patient at the first colonoscopy was analysed. In contrast, a higher number of patients were included in our study and moreover, all removed and available polyps from the baseline colonoscopy were analysed.

Surveillance colonoscopies are performed for polyps, due to the risk of developing advanced neoplasia. Recommendations for surveillance are based on different potential risk factors found in a baseline colonoscopy [33–34]. To date, the main known indicators of risk were polyp size, number, and a few pathological characteristics, such as the grade of dysplasia and the presence of a villous component [35–36]. In general, these recommendations are applicable to adenomas, but less evidence has supported follow-up recommendations for serrated lesions [37].

Molecular pathologic epidemiology is a relatively new field of epidemiology based on molecular classification of cancer that can help decipher interactions of environmental and lifestyle exposures with molecular pathology in cancer and premalignant tumors [38–39]. In the last few years, a classification system was developed for CRC molecular characteristics, based on *BRAF*, *KRAS*, and the CpG island methylator phenotype (CIMP) status, which could predict the prognosis and response to chemotherapy [19]. More recently, a comprehensive molecular classification of CRCs has also shown prognostic capability [40]. The present study was also designed along those lines, under the assumption that polyps, as precursor lesions for CRC, might also exhibit some early signatures of the pathway that could potentially lead to CRC. These pathway signatures could, at the same time, influence the risk of developing future lesions. Metachronous lesions that appear after polyp excision might develop under various conditions. On one hand, they might develop from missed or incompletely resected lesions, which might be related to the quality of the baseline colonoscopy. On the other hand, they may develop due to the biological characteristics of the lesions, which might promote rapid growth and progression to advanced states [41–42]. Both these possibilities could potentially explain the relationship between metachronous lesions and a carcinogenic pathway. For example, it is possible that *KRAS* mutated polyps might be more easily missed or incompletely resected than other types of polyps. Several reports have described the high risk of missing serrated lesions [43–46]; moreover, sessile serrated polyps were cited as a risk factor for incomplete endoscopic resection [47]. Although not all these lesions characteristically harboured *KRAS* mutations, a substantial proportion of *KRAS* mutated lesions were linked to the serrated pathway of carcinogenesis; thus, the difficulties in detecting and excising serrated polyps might, at least in part, apply to the association found here between *KRAS* mutations and the risk of developing advanced neoplasia. On the other hand, it is possible that *KRAS* mutated lesions might have a growth advantage. Moreover, it is also possible that a regional defect in the colon of patients that harboured *KRAS* mutated polyps might have exerted an effect that promoted the rapid development of these lesions after excision. Future studies should investigate all these potential explanations as well as potential relationships between molecular markers and lifestyle exposures in patients with colorectal polyps, following postulates of molecular pathological epidemiology [39].

Our study had several limitations. Importantly, it was a retrospective study, and our results must be confirmed with a prospective cohort, moreover it lacks a validation cohort that could confirm the results, avoiding potential selection bias. The study included only patients that received a second colonoscopy, and this population might not be completely representative of the general population. Another potential limitation was related to the definition of advanced serrated lesions. Currently, no standard definition has been established for advanced serrated lesions. This lack of definition hinders the formulation of a unified risk classification system for serrated polyps and adenomas. However, our findings provide information to support decisions about which polyps should be followed-up, due to the risk of developing advanced lesions and CRC, independent of polyp pathology. Very few studies have appropriately analysed risk factors for their ability to predict whether serrated polyps might develop into metachronous advanced lesions. Recommendations for the surveillance of these lesions varies among different guidelines. Some recent studies [15, 48] have shown that, among individuals with proximal, large serrated polyps, the risk of developing CRC is not lower than that of individuals with advanced adenomas. In this study, we adopted an arbitrary definition of advanced serrated polyps, based on previously recognised risk factors, including size, location, and the presence of dysplasia [15, 48–50]. Finally, we did not include analyses of the CIMP status or microsatellite instability (MSI) of polyps. CIMP, and particularly MSI, are late events in the serrated pathway of carcinogenesis; for the present study, we decided that, initially, we would study only early markers of the different carcinogenetic pathways. However, given our finding that *BRAF* would not be a useful marker for the risk of developing future advanced lesions, it is possible that adding CIMP status could improve our ability to characterise polyps in terms of risk.

In summary, this retrospective study was the first to find that *KRAS* mutations could play a potential role as a molecular marker for the risk of developing an advanced neoplasia during follow-up. These results should be confirmed in a prospective analysis, including a validation cohort. However, our findings could pave the way for going beyond size and number of lesions as main indicators for follow-up surveillance colonoscopies.
